# Effects on the Enthalpy of Microsynthesis Calorimetry of the Graft Copolymer Starch-g-Polycaprolactone for Five Starch Sources

**DOI:** 10.3390/polym17101311

**Published:** 2025-05-11

**Authors:** Noé Francisco Mendoza-Morales, Alejandro Aparicio-Saguilán, Delia E. Páramo-Calderón, Miguel A. García-Muñoz, Jesús Carrillo-Ahumada, José Eduardo Baéz-García, Javier Saldaña-Herrera, Enrique J. Flores-Munguía, Aurelio Ramírez-Hernández

**Affiliations:** 1 Centro de Investigaciones Científicas, Instituto de Química Aplicada, Universidad del Papaloapan, Circuito Central 200 Parque Industrial, San Juan Bautista Tuxtepec 68301, Oaxaca, Mexico; nmm2713f@gmail.com (N.F.M.-M.); garcia_m9@hotmail.com (M.A.G.-M.); 2 Ingeniería en Alimentos, Universidad del Papaloapan, Circuito Central 200 Parque Industrial, San Juan Bautista Tuxtepec 68301, Oaxaca, Mexicode_paramo@yahoo.com.mx (D.E.P.-C.); jesuscarrillo18@yahoo.com (J.C.-A.); 3 División de Ciencias Exactas, Departamento de Química, Universidad de Guanajuato, Noria Alta S/N, Guanajuato 36050, Guanajuato, Mexico; jebaez14@yahoo.com.mx; 4 Centro de Ciencias de Desarrollo Regional, Universidad Autónoma de Guerrero, Privada de Laurel No. 13, Col. El Roble, Acapulco 39640, Guerrero, Mexico; 19522@uagro.mx (J.S.-H.); enriqueflores@uagro.mx (E.J.F.-M.)

**Keywords:** starch, PCL, graft copolymer, enthalpy, thermal analysis

## Abstract

The aim of this work was to carry out a microsynthesis of a graft copolymer from different starch sources with polycaprolactone (PCL) and to evaluate its effects on enthalpy during synthesis via differential scanning calorimetry (DSC). The copolymer was characterized via FTIR and pasting profile techniques. FTIR studies revealed that starch–PCL graft copolymerization was carried out on all starch sources. The pasting profile revealed that the copolymer presented low viscosity values (heating and cooling stages), compared with those of native starches. This finding indicates that copolymerization took place on the surface of the starch granules. Cassava starch presented the highest enthalpy values at synthesis temperatures of 150 °C and 160 °C.

## 1. Introduction

Environmental pollution is one of the main problems worldwide, and conventional plastics play a significant role. Their production rate increases annually, and time it takes for them to degrade in the environment is estimated to be over 100 years. This has caused damage to various terrestrial and aquatic ecosystems [1]. Even though recycling methods exist, they are not sufficient, as the production rate exceeds the percentage that can be recycled through any of the existing methods. For this reason, in recent years, biodegradable polymers such as polycaprolactone (PCL) and starch have been investigated for their ability to synthesize a copolymer comprising a synthetic polymer and a natural polymer, respectively [2]. Polycaprolactone (PCL) is a biodegradable, semicrystalline polyester synthesized primarily via a ring-opening polymerization (ROP) reaction using initiators and catalysts such as Sn(Oct)_2_ and (NH_4_)_6_Mo_7_O_24_, respectively, among others [3,4,5,6]. It has been reported in the literature that the use of an ammonium heptamolybdate catalyst results in larger molar masses of the PCL polymer than other catalysts or initiators establish in less than two hours, and this catalyst can be easily separated and recovered from the final product [6].

PCL is a hydrophobic polymer that is soluble in organic solvents and can be degraded by acidic, basic, or aqueous hydrolysis [7,8]. PCL has attractive physical and chemical characteristics for mixing with other polymers and forming copolymers, and this polymer is not toxic to the human body; thus, it is used in biomedicine as a biomaterial [9]. PCL is a promising candidate for in situ tissue engineering due to its high mechanical load and slow degradation in the human body [10].

On the other hand, some biopolymers, such as starch, need to interact with other polymers in order to improve the functional and mechanical properties of the new materials that are obtained. PCL is one of the polymers used to provide these characteristics to starch. For example, starch-g-PCL graft copolymers have been synthesized from different starch sources, such as banana starch and corn starch [11,12,13].

In both copolymers, a more hydrophobic material with a higher modulus of elasticity was obtained compared to starch-only films. Ramírez-Hernández et al. [14] evaluated the effect of ethylene glycol on the synthesis of starch–PCL copolymers using molybdenum oxide as a catalyst. Infrared spectroscopy (FTIR) and nuclear magnetic resonance (NMR) analyses indicated that the copolymerization obtained a conversion yield of 84%. Ramírez-Hernández et al. [15] developed starch films by adding a starch–PCL graft copolymer and studied the physicochemical properties of the material. They reported that the moisture content and water vapor permeability decreased significantly. The crystallinity and elongation percentage of the films increased significantly with the addition of the copolymer. Chemical modifications to starch are important because, in recent years, there has been a search for natural materials that can compete with conventional plastics, which are not biodegradable and therefore increase environmental pollution when they end their useful life and are discarded in the environment. However, starch-only films have disadvantages, such as their high stiffness and affinity for water [16,17,18]. Therefore, it is necessary to obtain information related to these starch modifications to produce artificial plastics at the industrial level. The microsynthesis of PCL has been reported in the literature via differential scanning calorimetry, using ammonium decamolybdate as a catalyst to determine its enthalpy and polymerization model. However, the microsynthesis of starch–g–PCL graft copolymers by DSC using different starch sources has not been studied. Starch is a polysaccharide with a complex structure and shape that is found in cereals, legumes, tubers, and fruits. The physicochemical properties and chemical modifications of starch depend on several factors, such as its source and chemical composition, and the molar mass, shape, and size of the granule. Determining the copolymerization enthalpy of PCL with different starch sources will provide information on the chemical interactions between these two polymers.

Therefore, the objective of the present work was to evaluate the enthalpy behavior during the microsynthesis of starch–PCL graft copolymers via differential scanning calorimetry (DSC) using ammonium decamolybdate as a catalyst and different starch sources, such as banana, corn, cassava, taro, and mango. The ammonium decamolybdate catalyst was obtained in situ by heating ammonium heptamolybdate in the reaction medium.

## 2. Experimental Section

### 2.1. Materials

ε-Caprolactone (CL) (704067) and ammonium heptamolybdate tetrahydrate (NH_4_)_6_[Mo_7_O_24_] were obtained from Sigma-Aldrich (St. Louis, MO, USA). Corn starch, mango, banana, taro, and cassava were provided as gifts from the food laboratory of the University of Papaloapan.

### 2.2. Synthesis of the Graft Copolymer

Starch (1 mg) from each source was placed in an aluminum capsule approximately 10 mm in diameter, and ammonium heptamolybdate (1 mg) and CL (1 µL) were added to it. The capsule was then hermetically sealed with an aluminum cap via a mechanical press. The capsules were placed in the autosampler of the DSC equipment.

**Scheme 1:**
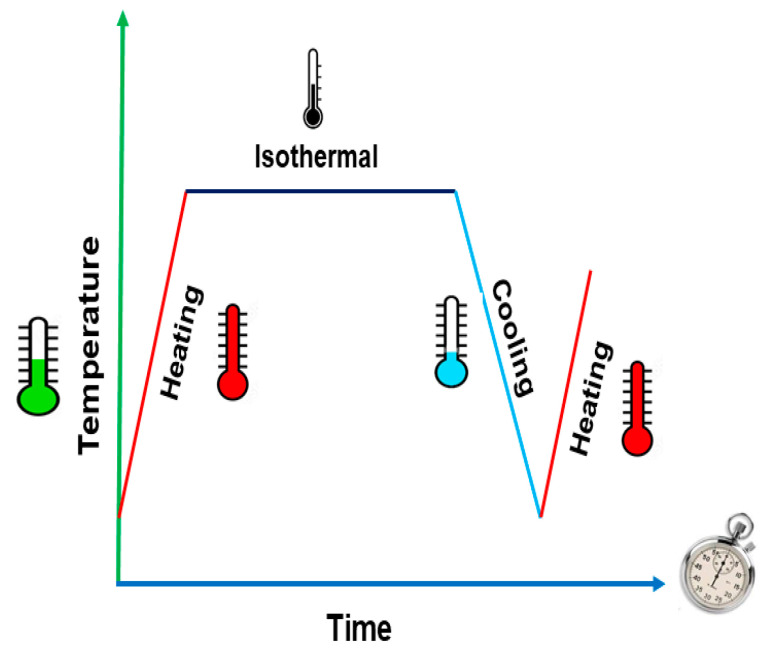
General method used in the synthesis of starch–g–PCL.

The process programmed in the DSC equipment is presented in Scheme 1 and consisted of three stages: heating from 25 °C to the copolymerization temperature (140 °C, 150 °C, and 160 °C) at a rate of 10 °C/min, followed by an isothermal process at the copolymerization temperature for 180 min; subsequently the sample was cooled until it reached room temperature and then subjected to a heating process at 25 °C until reaching the temperature of 100 °C at a rate of 10 °C/min. In this last stage of the process, a thermogram of the synthesized copolymer was obtained.

### 2.3. Characterization of Starch–g–PCL

#### 2.3.1. DSC Measurements

The synthesis and characterization of the starch–g–PCL copolymer were studied using a TA Instruments (model DSC 250, New Castle, DE, USA) differential scanning calorimeter. The methodology reported by Ramirez-Hernandez for the polymerization of ε-CL was modified [19]. In the present paper, nitrogen was used as the purging gas to avoid the effect of oxygen on the copolymerization reaction in the reaction medium.

The transitions observed in the thermograms were analyzed using the TA Instruments software TRIOS v4.02.30774.

#### 2.3.2. Analysis of Starch–g–PCL by Fourier Transform Infrared Spectroscopy

Infrared spectra of the films were recorded at room temperature in a Perkin-Elmer Spectrum 100FT-IR (ATR) spectrometer (Shelton, CT, USA) with a resolution of 4 cm^−1^ and averaged over 16 scans in the range of 4000–650 cm^−1^.

#### 2.3.3. Pasting Properties

The pasting profile was obtained using a 10% (*w*/*v*) dispersion in a hybrid rheometer (TA Instruments), model HR-2. The dispersion was subjected to a heating–cooking–cooling cycle. The initial temperature was 30 °C, which was maintained for 60 s then heated to 90 °C at 15 °C/min; this temperature was maintained for 10 min (cooking) before finally being cooled to 30 °C at 30 °C/min; this temperature was maintained for 7 min. The software 5332-1679 TA Instruments TRIOS v4.02.30774 was used to obtain the paste temperature, peak time, and viscosity peak.

#### 2.3.4. Statistical Analysis

The results obtained regarding the physical properties of the films were subjected to a one-way analysis of variance (ANOVA), followed by Tukey’s test. If the analysis showed significant differences (*p* < 0.05), the means were compared using Tukey’s test at a level of significance of 0.05. Statistical analyses were performed using the SPSS V.6.0 software (SPSS Institute Inc., Cary, NC, USA).

## 3. Results and Discussion

### 3.1. Synthesis of the Graft Copolymer

The reaction between PCL and starch occurred primarily through an esterification reaction due to the functional groups present in both polymers. It is worth mentioning that the polymerization of caprolactone primarily takes place on the surface of the starch granules. The presence of the ammonium molybdate catalyst used during the capralactone polymerization process on starch granules has no effect on the synthesized copolymer because this catalyst can be separated using organic solvents such as chloroform [14]. Furthermore, the presence of the catalyst does not lead to a signal in the midrange of the FTIR spectrum and does not influence the pasting analysis of the synthesized copolymer due to its concentration in the formulation of the chemical reaction. Furthermore, it does not affect the DSC analysis due it does not undergo any chemical or physical transitions to obtain the copolymer thermogram in the studied temperature range. The only transition the catalyst would be expected to undergo would be its chemical decomposition, which would occur above 700 °C.

**Figure 1:**
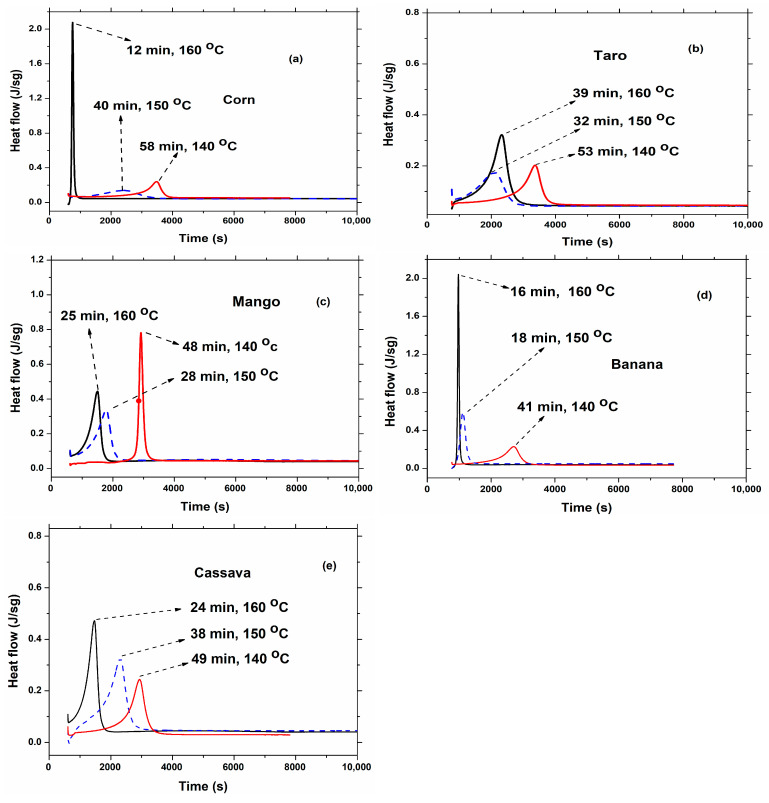
Thermograms of the synthesis of the starch–g–PCL graft copolymer. Corn starch–g–PCL (**a**), taro starch–g–PCL (**b**), mango starch–g–PCL (**c**), banana starch–g–PCL (**d**) and cassava starch–g–PCL (**e**).

The starch–g–PCL graft copolymer was synthesized in situ via differential scanning calorimetry at three temperatures with five starch sources. Figure 1 presents the thermograms of the synthesized copolymer, as well as the curve representing the enthalpy of the copolymerization reaction.

In each of the thermograms, the observed processes are endothermic; that is, these processes require energy to take place.

At a synthesis temperature of 160 °C, the five starch sources required the shortest times to reach the peak of the maximum reaction enthalpy. This behavior was expected because the reaction rate increases when the reaction temperature increases and, consequently, the reaction time decreases [20,21]. In the literature, it has been reported that caprolactone polymerization occurs within two hours via heterogeneous catalysis in the temperature range of 150 °C to 160 °C. At these temperatures, no transesterification or backbiting reactions are detected. However, it has been reported in the literature that at a temperature of 160 °C, the polymerization reaction is very rapid, and the molecular weight cannot be controlled [19]. The corn starch (Figure 1a) source reached a copolymerization time of 12 min, while the taro starch (Figure 1b) took the longest time (39 min). This difference in time is mainly due to the differences in the chemical composition between these starch sources, the differences in the structural characteristics of the starch components (amylose/amylopectin), and the morphology of the starch granule. In corn starch, corn granules have circular and polygonal morphologies, as reported in the literature [22,23]. The morphology of taro starch granules is mainly polygonal [24,25]. Therefore, circular starch granules more easily undergo chemical modification than polygonal granules due to the contact area between the reactants [26,27,28]. This difference in morphology has a greater impact on the reaction time than the granule size because taro starch has a smaller granule size than corn starch, and it is expected that the smaller the granule size, the easier its modification, as reported in the literature [23,29,30].

Furthermore, corn starch has a lower lipid and protein content compared to taro starch, which directly affects its processing of starch and its reactions with other components or substances [24,31]. In this case, PCL could react with the lipids and proteins present inside the starch granule, and PCL homopolymerization is favored over copolymerization. However, there is also the possibility that at temperatures above 100 °C, the starch granules may begin to partially or completely degrade. All these factors affect its selectivity, causing it to react only with PCL and starch, which directly impacts the yield, reaction time, and thermal behavior of the synthesized copolymer.

In addition, corn starch contains a greater proportion of amylose than taro starch (17.45% vs. 9.41%). Amylose facilitates the chemical modification of starch through the number of OH groups available in the monomeric unit of starch [14,32].

The hydroxyl (OH) group content favors chemical and physical modifications of starch. Furthermore, starch contains three OH groups in its monomeric unit at carbons C2, C3, and C6, which could undergo a grafting reaction with PCL [33]. However, C6 of starch presents less steric hindrance, making it more likely that PCL will bond with starch at this carbon [34]. This variable composition is clearly observed in plantain starch (Figure 1d), which has a reaction time of 16 min (Figure 1a–c,e). Some authors carried out the polymerization of polycaprolactone (PCL) by means of DSC, with a reaction time of 20 min [19]. This homopolymerization time is longer than that required for copolymerization, which means that starch facilitates the reaction. The copolymerization enthalpy was determined from the area under the curve of the signal observed in the thermogram. This was obtained via DSC software (TRIOS v4.02.30774). Table 1 presents the enthalpy values of the synthesis of the starch–g–PCL copolymer, as well as its melting temperature, for each of the copolymerizations that were carried out.

From the results of the reaction enthalpy, a relation can be observed between its value and the size of the starch granule; when the size increases, the enthalpy is lower and vice versa, with banana starch having the largest granule size compared with the other four sources [35]. This starch has the lowest enthalpy value compared to the other starches (118 J/g) at a copolymerization temperature of 160 °C. The enthalpy value is directly related to the absorption (endothermic) or release (exothermic) of heat by a substance, but is also related to the reaction time. In the case of the starch–g–PCL graft copolymer synthesis, the enthalpy value of each starch source varies due to various factors, such as its chemical composition, particle size, morphology, and molar mass. Cassava starch could obtain a value of 199.4 J/g, which was the highest compared with the other sources studied in this research, at a temperature of 150 °C; the second highest value was by mango starch. However, the copolymerization times of these two starch sources differed by 10 min. This indicates that the activation energy needed to convert reactants to products is likely reached more quickly in mango starch than in cassava starch (28 min vs. 38 min). It is worth noting that when the temperature is reduced to 140 °C, the times are longer than at other synthesis temperatures. In this research, it is difficult to observe a relationship between time and starch source that would allow for the prediction of any enthalpy trend or behavior. Determining the enthalpy in the chemical reaction of starch using the in situ polymerization of copralactone on the surface of the starch granule is important as this can provide a foundation for copolymerization reactions between a natural and synthetic polymer and improve their viability compared with conventional nonbiodegradable plastics. Graft copolymerization enthalpies were reported for other polymers via DSC, such as the chitosan–graft–PEHA copolymer (800 kJ/mol), but, until now, no information has been reported on starch–g–PCL copolymers [36]. The melting temperature of PCL in the copolymer depends on the synthesis temperature of the copolymer; when this temperature decreases, the temperature Tm decreases. Values of up to 57 °C can be reached for the corn and banana sources when a synthesis temperature of 160 °C is used, whereas at a synthesis temperature of 140 °C, the temperature Tm reaches 44 °C and 37.6 °C, respectively.

### 3.2. FTIR Analysis

**Figure 2:**
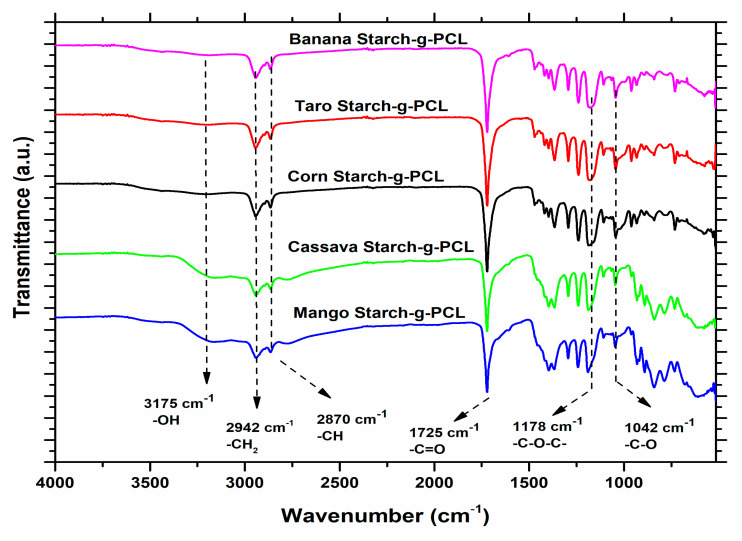
FTIR spectra of starch–g–PCL graft copolymers at 160 °C.

The infrared spectra of each of the copolymers are presented in Figure 2. Characteristic signals of starch vibration were observed in all infrared spectra.

The signal corresponding to the stretching vibration of the hydroxyl groups (OH) of starch appears in the range 3175 cm⁻^1^–3220 cm⁻^1^. Another characteristic signal of starch appears in the range of 1178 cm⁻^1^–1042 cm⁻^1^, which corresponds to the bending vibration of the C-O-C group present in its monomeric structure. The signal in the range of 900 cm⁻^1^–928 cm⁻^1^ corresponds to the vibration signal of the α(1–6) bond. The main vibration signals of PCL in the starch–g–PCL copolymer were observed in each of the spectra. The stretching vibration signal of the hydroxyl group (OH) of PCL overlaps with the vibration signal of the hydroxyl group of starch in the range of 3159 cm⁻^1^–3220 cm⁻^1^. The vibration signal of the carbonyl group (C=O) present in the monomeric unit of PCL appears in the range of 1718 cm⁻^1^–1725 cm⁻^1^. This signal also appears in all the FTIR spectra of the copolymers when using different temperatures for the synthesis of the copolymers. It is worth mentioning that the vibration signals of the methylene group (CH_2_) of both polymers appear in the range of 2939 cm⁻^1^–2944 cm⁻^1^, and this signal was observed in all the copolymerization spectra. The differences in the vibration signals in the range of 1360 cm⁻^1^ to 1470 cm⁻^1^ between the cassava and mango spectra with respect to those of the other three starch sources are mainly due to their chemical compositions.

For example, according to what has been reported in the literature, mango starch and cassava starch have a higher water content and lower protein contents than the other three starch sources studied in this work, which influences the synthesis of the starch–g–PCL graft copolymer [37].

In the FTIR spectra of mango starch and cassava starch, there is a marked difference in the region, called the fingerprint, which covers the range of 600 cm⁻^1^ vs. 1000 cm⁻^1^, and that of the other three starch sources. The width of the signals observed in these two FTIR spectra indicates that their chemical composition contains inorganic or mineral compounds at higher concentrations than those in the other three starch sources. In addition, a notable difference can also be observed in the area of vibration of the hydroxyl groups (OH); in these spectra, it is possible to observe a weak vibration signal at 3500 cm⁻^1^. These signals may be due to the presence of proteins or lipids on the starch surface, which were not observed in the three starches. The conformation or arrangement of all the chemical components present in the starch is important to determine in order to explain the physicochemical and mechanical behavior of materials obtained from this natural polymer. The starch itself would have a different physical/chemical compartment than the PCL-modified starch.

Therefore, it can be said that the vibration signals of the starch and PCL in the copolymer agree with the signals reported by other researchers, and this indicates that PCL was polymerized on the surface of the starch granule to obtain the graft copolymer starch–g–PCL [14].

**Scheme 2:**
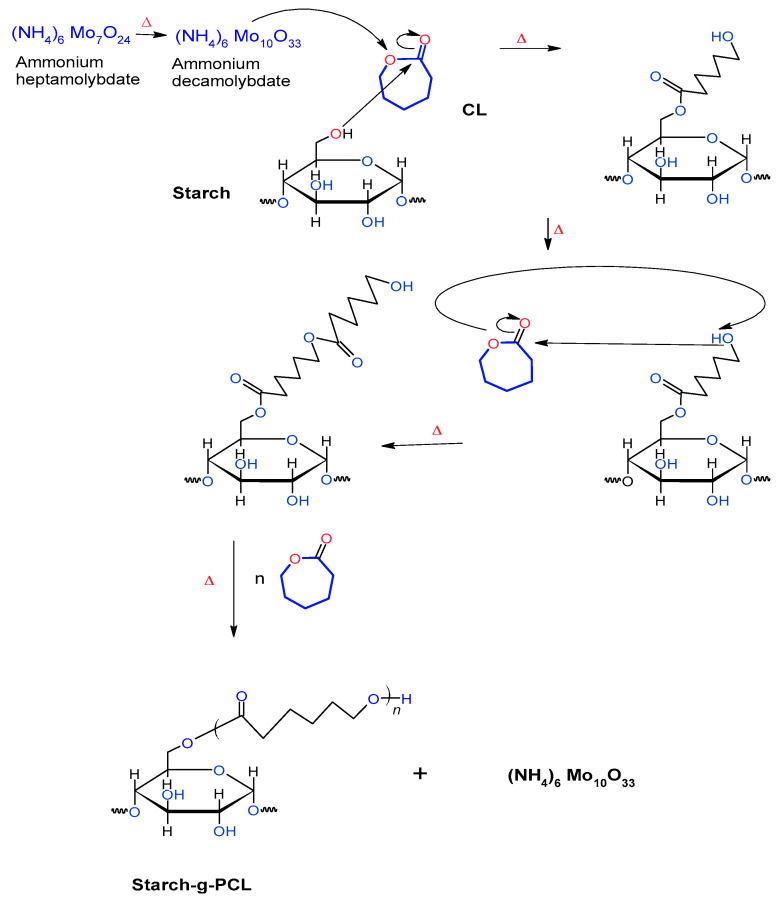
Proposed mechanism of the copolymerization of starch with PCL.

The copolymerization mechanism of starch with PCL is presented in Scheme 2.

In Scheme 2, the copolymerization reaction occurred in C6 of starch and the carbonyl group of caprolactone. The ammonium heptamolybdate catalyst was first converted in situ to ammonium decamolybdate through heating to promote ring-opening in the CL.

### 3.3. Starch–g–PCL Paste Formation Process

The starch-pasting analysis carried out in the present investigation allowed for an evaluation of the paste-forming properties of starches through heating a starch suspension and measuring its viscosity.

**Figure 3:**
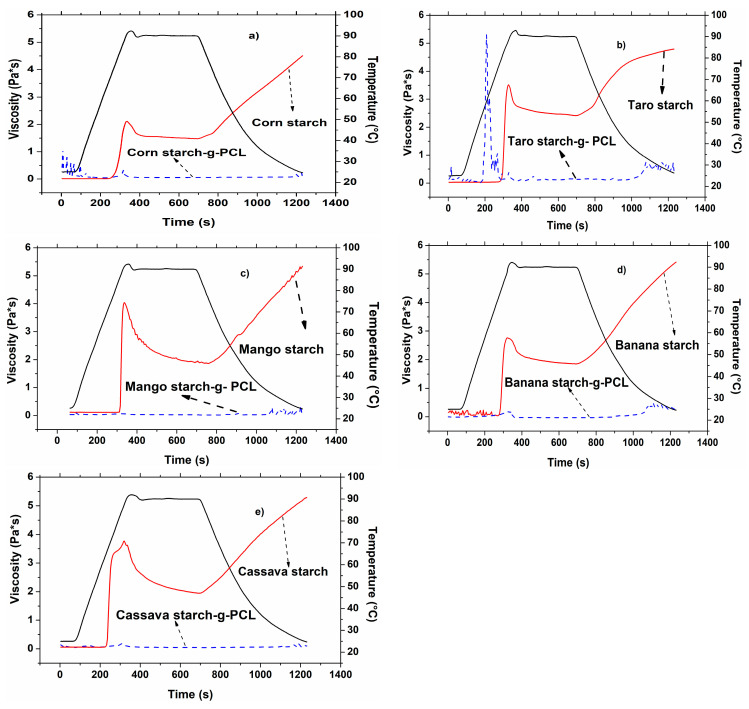
Pasting analysis of the starch–g–PCL graft copolymer. Corn starch–g–PCL (**a**), taro starch-g-PCL (**b**), mango starch–g–PCL (**c**), banana starch–g–PCL (**d**), and cassava starch–g–PCL (**e**).

The graphs of the viscosity behavior of native and modified starches from each of the sources with respect to temperature and time are shown in Figure 3.

From these pasting curves, a tendency toward a decrease in maximum viscosity can be observed in all modified starches; that is, the maximum viscosity in the heating process and the viscosity in the cooling stage are lower than those of native starches. This effect is due to the interaction of the PCL chains on the surface of the starch granule, which is incorporated and prevents the passage of water molecules into the granule, thereby inhibiting gelatinization and the subsequent leaching of amylose. During the pasting process, amylose chains diffuse into the water, forming a gel, while amylopectin loses its crystalline order. This gelatinization process, or order–disorder transition, that starch polymers undergo when heated impacts the processing and stability of starch-based products.

Furthermore, in the gelatinization process, the molecular order within the granules, which is mainly associated with amylopectin, is gradually and irreversibly destroyed. Therefore, the gelatinization temperature is characteristic of each type of starch and fundamentally depends on the glass transition of the amorphous fraction of the starch and the amylose/amylopectin composition.

These results agree with those obtained via FTIR and DSC, as described in other studies [14]. Changes in the viscosity profile of starches are influenced by the different physicochemical and chemical changes that occur during their copolymerization with PCL.

However, this behavior was not observed for taro starch, since its viscosity was higher compared to that of native starch (Figure 3b). These results are due to the fact that the granular structure of taro starch was affected by the reaction conditions described in the synthesis methodology, allowing for water molecules to be incorporated in greater quantities than in native starches.

The viscosity vs. temperature diagram of native taro starch shows a lower viscosity value at room temperature compared to the other sources (Figure 3a,c,d,e); however, this starch source presented the greatest instability during the pasting test. Moreover, mango and cassava (Figure 3c,e) starches had the greatest stability during this test. This is probably due to the fact that the interaction between the starch granules and the PCL is more uniform, and the distribution of the PCL polymer chain over the starch surface is greater compared to that of the other starch sources.

These results indicate that starch–g–PCL presents hydrophobic characteristics, provided by the PCL chains, and also characteristics such as the starch being resistant to aqueous hydrolysis conditions. This is due to the reduction in hydroxyl groups present in the starch structure due to its chemical interaction with the PCL chains. Furthermore, this characteristic favors its potential application as a material compatible with hydrophobic substances and its stability in film formation. In recent years, there has been a search for environmentally friendly polymeric materials for use as flocculants in wastewater purification [38], and given the chemical and physical characteristics of the synthesized copolymer, it could be used for this purpose.

Additionally, this copolymer has strong potential to agglomerate the microplastics present in drinking water, which represents an opportunity since many of the materials used to achieve microplastic agglomeration are toxic to the environment and to humans. Furthermore, the five starch sources studied in this research also provide a comparison between conventional and nonconventional starches, increasing our understanding of the physical and chemical complexity of starches.

## 4. Conclusions

The synthesis of a starch–g–PCL graft copolymer was carried out with DSC equipment, using five starch sources. On the basis of the reaction conditions proposed in this research, a temperature of 140 °C was deemed the most suitable to carry out the synthesis of the graft copolymer. At this temperature, a melting peak related to PCL was observed in the thermograms, whereas, at higher temperatures, several peaks were observed, which indicates the generation of polymer chains of different sizes. The reaction enthalpies were determined for each of the sources, revealing an increasing trend with respect to the copolymerization temperature.

In this study, the activation time required for the monomer to be converted to a polymer was found to be proportional to the amylose content. Plantain starch was observed to have the most favorable proportion, with a shorter activation time observed in the monomer. FTIR characterization verified that the graft copolymer was obtained. In the FTIR spectrum of this copolymer, the typical vibration signals of PCL and starch were observed.

A paste analysis of the copolymer elucidated the viscosity of the starch. The viscosity was affected by a drastic decrease in the graft copolymer because the PCL chains grafted onto the granule do not allow the granule to absorb water molecules and undergo gelatinization.

## Figures and Tables

**Table 1:** Synthesis enthalpy and melting temperature of starch–g–PCL.

Source	Synthesis Temperature (°C)	Time (min)	Enthalpy (J/g)	Tm (°C)
Banana	160	16	118.0	57.0
	150	18	112.9	57.0
	140	41	130.7	44.0
Taro	160	39	179.8	45.6
	150	32	104.7	37.4
	140	53	108.2	35.6
Corn	160	12	153.6	57.3
	150	40	113.4	45.0
	140	58	108.1	37.6
Cassava	160	24	163.1	40.4
	150	38	199.4	44.0
	140	49	110.1	39.4
Mango	160	25	151.7	56.8
	150	28	151.5	39.3
	140	48	119.9	39.3

## Data Availability

The original contributions presented in this study are included in the article. Further inquiries can be directed to the corresponding author.
